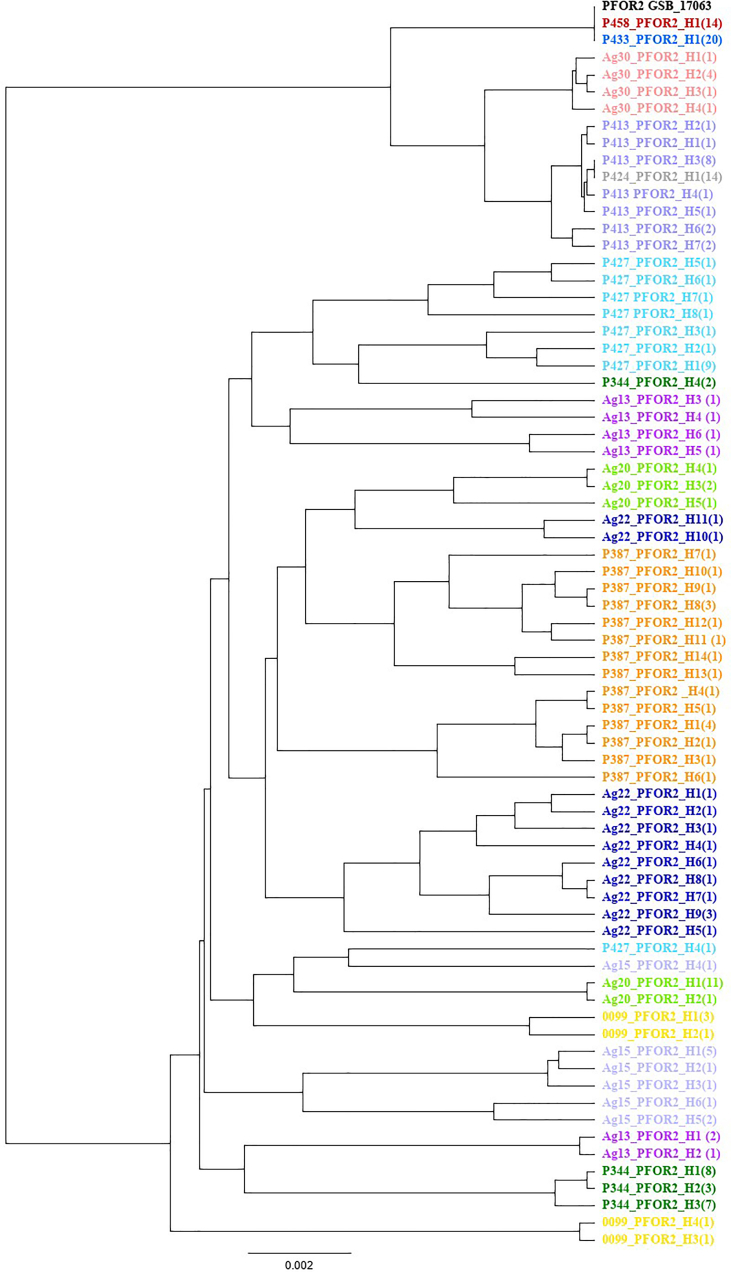# Genetic diversity in the metronidazole metabolism genes nitroreductases and pyruvate ferredoxin oxidoreductases in susceptible and refractory clinical samples of *Giardia lamblia*

**DOI:** 10.1016/j.ijpddr.2022.12.003

**Published:** 2022-12-21

**Authors:** Christina S. Saghaug, Astrid L. Gamlem, Kirsti B. Hauge, Juha Vahokoski, Christian Klotz, Toni Aebischer, Nina Langeland, Kurt Hanevik

**Affiliations:** a Department of Clinical Science, University of Bergen, Bergen, Norway; b Norwegian National Advisory Unit on Tropical Infectious Diseases, Department of Medicine, Haukeland University Hospital, Bergen, Norway; c Department of Medicine, Haukeland University Hospital, Bergen, Norway; d Department of Infectious Diseases, Unit 16 Mycotic and Parasitic Agents and Mycobacteria, Robert Koch-Institute, Berlin, Germany

**Keywords:** Nitroreductase, Pyruvate ferredoxin oxidoreductase, Metronidazole, Genetic diversity, Allele, Resistance, SNP, SNV, Nonsense mutation, MTZ, Metronidazole, SNV, Single nucleotide variant, nsSNV, non-synonymous SNV, NR, nitroreductase, PFOR, pyruvate ferredoxin oxidoreductase

## Abstract

The effectiveness of metronidazole against the tetraploid intestinal parasite *Giardia lamblia* is dependent on its activation/inactivation within the cytoplasm. There are several activating enzymes, including pyruvate ferredoxin reductase (PFOR) and nitroreductase (NR) 1 which metabolize metronidazole into toxic forms, while NR2 on the other hand inactivates it. Metronidazole treatment failures have been increasing rapidly over the last decade, indicating genetic resistance mechanisms. Analyzing genetic variation in the PFOR and NR genes in susceptible and refractory *Giardia* isolates may help identify potential markers of resistance.

Full length *PFOR1*, *PFOR2*, *NR1* and *NR2* genes from clinical culturable isolates and non-cultured clinical *Giardia* assemblage B samples were cloned, sequenced and single nucleotide variants (SNVs) were analyzed to assess genetic diversity and alleles.

A similar ratio of amino acid changing SNVs per gene length was found for the NRs; 4.2% for *NR1* and 6.4% for *NR2*, while the *PFOR1* and *PFOR2* genes had less variability with a ratio of 1.1% and 1.6%, respectively. One of the samples from a refractory case had a nonsense mutation which caused a truncated *NR1* gene in one out of six alleles. Further, we found three *NR2* alleles with frameshift mutations, possibly causing a truncated protein in two susceptible isolates. One of these isolates was homozygous for the affected *NR2* allele. Three nsSNVs with potential for affecting protein function were found in the ferredoxin domain of the *PFOR2* gene. The considerable variation and discovery of mutations possibly causing dysfunctional NR proteins in clinical *Giardia* assemblage B isolates, reveal a potential for genetic link to metronidazole susceptibility and resistance.

## Highlights

• Mutations in ferredoxin domains of thr nitroreductases and pyruvate ferredoxin oxidoreductases may alter protein function.
• Nonsense mutation in nitroreductase 1 alleles may contribute towards metronidazole tolerance in *Giardia*.
• Susceptible *Giardia* isolates had frameshift mutations in nitroreductase 2 alleles, possibly affecting metronidazole toxicity.

## Graphical abstract

**Figure:**
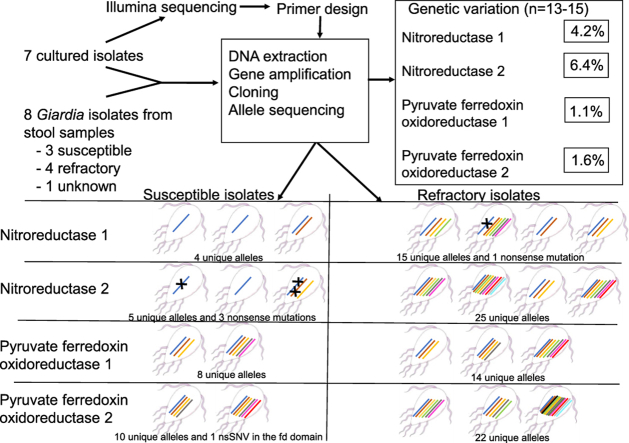

## 1 Introduction

*Giardia lamblia* is a flagellated microaerophilic protozoan parasite causing asymptomatic or symptomatic intestinal infection worldwide with almost 300 million clinical cases of *Giardia* being reported annually (Ankarklev et al., 2010; Kirk et al., 2015). The species *G. lamblia* is divided into eight distinctive assemblages, where assemblages A and B are known to infect humans, the latter being more frequently reported in human infections (Caccio and Ryan, 2008; Ankarklev et al., 2010). Assemblage B isolates usually show a higher genetic diversity and allelic sequence heterozygosity (ASH) than assemblage A isolates (0.5% vs. <0.01–0.04%) (Yu et al., 2002; Franzen et al., 2009; Ankarklev et al., 2012).

The first-line treatment against giardiasis, in most countries, is the prodrug metronidazole (MTZ), with an estimated efficacy rate ranging from 60% to 100% (Argüello-García et al., 2020). However, over the past decade, treatment failures with MTZ have been reported more frequently and has become a growing concern (Lalle and Hanevik, 2018), with 15–70% of the cases not cured with a standard 5–7-day course of MTZ treatment (Munoz Gutierrez et al., 2013; Nabarro et al., 2015; Requena-Mendez et al., 2017; Cañete et al., 2020; Ydsten et al., 2021; Neumayr et al., 2021).

MTZ needs to be activated intracellularly by a partial reduction at its nitro group, in order to create highly reactive toxic intermediates (Leitsch, 2015). MTZ has previously been demonstrated to be activated by three enzymes in *Giardia*, namely the nitroreductase (NR)1 (Pal et al., 2009), pyruvate-ferredoxin oxidoreductase (PFOR)1 (Leitsch et al., 2011) and the thiol-cycling associated enzyme thioredoxin reductase (Leitsch et al., 2016). NR2 inactivates MTZ by fully reducing it to an inert and non-toxic form of the antibiotic (Muller et al., 2013, 2015).

There are two PFOR genes in *Giardia*, named PFOR1 and PFOR2. The PFORs together with the co-factor ferredoxin serve as initial enzymes in electron transport reactions in *Giardia* i.e., activation of MTZ (Townson et al., 1996; Leitsch et al., 2011; Ansell et al., 2017). PFOR1 has previously been inhibited by a hammerhead ribozyme, showing that reduced expression of PFOR1 rendered *Giardia* more resistant towards MTZ and caused better growth in the presence of oxygen (Dan et al., 2000). Even if lower levels of PFOR1 gene expression have been linked to MTZ resistance, it has not yet been found to be significantly different between susceptible and resistant isolates, and thus not deemed a good marker of MTZ resistance (Begaydarova et al., 2015).

The NRs are essential metabolic enzymes that most likely have been acquired by lateral transfer from anaerobic bacteria or archaebacteria (Nixon et al., 2002; Muller and Muller, 2019). The two different NRs in *Giardia*, NR1 and NR2 are considered to be paralogs with different MTZ metabolizing capacities (Muller et al., 2013, 2015; Ansell et al., 2017). NR1 expression in MTZ-resistant laboratory *Giardia* lines has been found to be downregulated, whereas NR2 has been found to be upregulated (Leitsch, 2019). Further, *E. coli* expressing both NR1 and NR2 from *Giardia* has been shown not to be susceptible to MTZ, indicating that NR2's MTZ inactivating capabilities surpass NR1's activating ones (Muller et al., 2015).

There are currently no known genetic markers of MTZ resistance in *Giardia*, and genetic diversity represented by allele identification and characterization by cloning of the whole CDS has only been carried out for a handful of studies (Lasek-Nesselquist et al., 2009; Kosuwin et al., 2010; Siripattanapipong et al., 2011; Choy et al., 2015; Aguiar et al., 2016; Gabin-Garcia et al., 2017; Mizuno et al., 2020). Most studies of MTZ resistance in *Giardia* have examined cultivable and historical strains of sub-assemblage AI (WB) which rarely infect humans and may not be representative for sub-assemblage AII and assemblage B normally infecting humans (Caccio and Ryan, 2008). In these studies, MTZ tolerance has been induced by slowly increasing MTZ concentrations in growth medium, meaning they are not naturally resistant. Various potential adaptive mechanisms have been identified (Ansell et al., 2017; Müller et al., 2018; Muller et al., 2008). However, these adaptations seem to be lost after one en-/excystation cycle, indicating that such acquired resistance is not necessarily passed on from one infection to the next (Muller et al., 2008). Still, the rapid increase of treatment refractory *Giardia* infections, especially those seen over the last decade in South Asia, point towards heritable traits enabling *Giardia* to resist MTZ treatment.

The aim of this study was to analyze genetic variation and allelic composition of *Giardia* PFOR1 and 2 and NR1 and 2 in recently axenized and in non-cultured assemblage B clinical isolates. Some of the isolates were obtained from MTZ treatment refractory patients. The identified genetic variants were evaluated in relation to clinical information where this was available and for their potential effect on protein function.

## 2 Materials and methods

### *2.1* *Giardia* samples, purification, DNA extraction and PCR

Two different sets of *Giardia* clinical samples were used in the present study. One set represents trophozoite cultures of a *Giardia* biobank at the Robert Koch-Institute (RKI) in Berlin, with seven *Giardia* assemblage B isolates that were cultured as previously described (Keister, 1983). The other set represents DNA from non-cultured MTZ susceptible or MTZ refractory clinical samples of *Giardia* assemblage B infections collected from 2004 to 2017 at Haukeland University Hospital, Bergen, Norway. Trophozoites from the RKI culturable isolates were collected followed by DNA extraction and concentration measurements before Illumina whole genome sequencing was carried out according to (Saghaug et al., 2019). No clinical data were available for these *Giardia* isolates.

The cysts from the non-cultured isolates were obtained from 19 returning travelers. The cysts were purified from patient stool samples by sucrose flotation alone or in combination with immunomagnetic separation (IMS) (Dynabeads® G-C combo kit, Life technologies) according to (Hanevik et al., 2015) but omitting the final steps releasing cysts from beads. Cysts from a sample originating from the 2004 Bergen outbreak, VA, was obtained by salt flotation and IMS and DNA was extracted according to (Robertson et al., 2006).

DNA from the isolated *Giardia* cysts was extracted using the kit MagAttract® HMW DNA (Qiagen). *Giardia* cysts with beads from previous IMS steps, were frozen and thawed three times before adding proteinase K followed by AL buffer and RNase A from the MagAttract® kit. The samples were then incubated at 56 °C for 1 h according to (Ogren et al., 2016), followed by 2 freeze-thaw cycles before a final heat incubation at 98 °C for 15 min. Remaining beads and cyst wall debris were pelleted and removed by *centrifugation* at 11000xG for 2 min before following the manufacturer's instructions.

qPCR detection of *Giardia* was carried out for the 19 non-cultured, purified cyst samples with the Light Cycler 480 II instrument (Roche *Diagnostics* GmbH, Mannheim, Germany), according to (Yang et al., 2014), except for using a *Giardia* assemblage B-specific forward primer, FAM probe was used instead of Joe 670 and a standard curve using 10 fold dilutions of a quantified pUC57 based plasmid construct (Genscript, NJ, USA) containing the target sequence of glutamate dehydrogenase (*gdh*), accession number MT108431.1. The primers and probes are listed in Table 1.

**Table 1:** **Primer pairs for glutamate dehydrogenase qPCR, nitroreductase 1 and 2 genes, pyruvate ferredoxin oxidoreductase 1 and 2 amplification PCR and pyruvate ferredoxin sequencing primers.** The *gdh* primers obtained from (Yang et al., 2014), with adapted assemblage B specific forward primer and a different quencher for the probe. The primers for the *NR* genes were designed using Geneious prime, and specificity was checked using the NCBI blast tool. Primer design was based on pre-and post-CDS conserved regions derived from eight previously Illumina sequenced cultured assemblage B isolates. For the *NR1*, two different primer sets were used in order to obtain positive PCR reactions for all isolates. All primers ordered from TIB Molbiol, Berlin, Germany.

Gene	Direction	Primer Sequence (5’ – 3′)	Bp	[Tm]^a^	Position related to CDS	Amplicon length	Reference
***gdh***	Forward	GGGCAAGTCGGACAACGA	18	61	−434	262 bp	(Yang et al., 2014) and this study
***gdh***	Reverse	GTCTACTTCCTGGAGGAGATGTGC	24	62.2	−696		Yang et al., (2014)
***gdh***	Probe	6FAM-TCATGCGCTTCTGCCAG-BBQ	17	62.3	−454		Yang et al., (2014)
***NR1***	Forward	GTGATGGAGCAAAGTCGC	18	64 °C	−162	1135 bp	This study
***NR1***	Reverse1	GTGGATGGGGCTCTTGAATA	20	64 °C	+178		This study
***NR1***	Reverse2	GTGGATGAGGCTCTTGAATA	20	61 °C	+178		This study
***NR2*.1**	Forward	ATCTACATAAGATCCGCGCACT	22	65 °C	−174	1212 bp	This study
***NR2*.1**	Reverse	TACTCTGCACCTCATCGCCG	20	69 °C	+171		This study
***NR2*.2**	Forward	GACTCACAGAGTGGCAACGA	20	67 °C	−251	1271 bp	This study
***NR2*.2**	Reverse	CGCCGAGCAATGTAGTGGTT	20	68 °C	+156		This study
***PFOR1***	Forward	CACAGTCCCCAATCACAGAC	20	59 °C	−143	3987 bp	This study
***PFOR1***	Reverse	GGAGGACATGGAGAGCAAGG	20	61 °C	+82		This study
***PFOR1***	Sequencing1	TGCCAAGTGGAGGAGCGAAA	20	59 °C	NA	NA	This study
***PFOR1***	Sequencing2	AGCACACGCCACATCGAG	18	58 °C	NA	NA	This study
***PFOR1***	Sequencing3	GACAAGTACGTCAAGGACATCA	22	58 °C	NA	NA	This study
***PFOR1***	Sequencing4	CACTGAGAGCTGCAACCTC	19	58 °C	NA	NA	This study
***PFOR2***	Forward	CGCATAATAGCTTTGACCGTT	21	55 °C	−66	4018 bp	This study
***PFOR2***	Reverse	TTCCAGCTTCTGTCGCTAC	19	56 °C	+152		This study
***PFOR2***	Sequencing1	TCCGATCTGGATGCAGGC	18	58 °C	NA	NA	This study
***PFOR2***	Sequencing2	GTCTCTTCTTCGGAATGGGATCC	23	62 °C	NA	NA	This study
***PFOR2***	Sequencing3	AAGGTCGTCAACATGAACCTTG	22	58 °C	NA	NA	This study
***PFOR2***	Sequencing4	TGCCAGGTCTGCTCCCAG	18	61 °C	NA	NA	This study

NA= Not applicable.

a Tm is estimated from NEB T_m_ calculatorwith 400 nM primer concentration and Q5® polymerase.

Isolates with ∼900 gene copies per μl sample, or higher, were selected for downstream experiments, resulting in a total of eight samples. The eight samples included one sample from an individual where treatment result was not known, three samples from *individuals* clinically MTZ susceptible and four from MTZ refractory individuals. Sample overview can be found in the Supplementary Table 1. Samples with fractionated DNA, contaminants, inhibitors or low concentrations of *Giardia* DNA were not included in the analysis.

### 2.2 Cloning the genes

Genomic *Giardia* DNA was used in polymerase chain reactions (PCR) for obtaining the *PFOR1*, *PFOR2, NR1* and *NR2* genes. All gene-PCRs were carried out using the Q5® High-Fidelity DNA Polymerase (catalog nr M0491L, New England BioLabs (NEB) Ipswich, MA, USA) and reaction setup for 25 μl according to manufacturer. Negative controls with nuclease free water were included for each experiment. Primers used to obtain full length genes are shown in Table 1. The gene names of the NRs in the current study are based on two publications by Müller et al. from 2007 to 2013, where *NR1* (GSB_22677) is also known as *GlNR1* or *Fd-NR2* in the GiardiaDB database, while *NR2* (GSB_153178) is also known as *GlNR2* or *Fd-NR1* (Muller et al., 2007, 2013).

The following PCR conditions were used: an initial denaturation of 98 °C for 30 s, 35–40 cycles of 98 °C for 10 s, 62–64 °C for 20 s and 72 °C for 31 s, followed by a final extension of 72 °C for 2 min. 20 μl PCR products were run on a 1% agarose gel pre-stained with GelRed® Nucleic Acid Stain (catalog nr: 41003, Biotium, San Francisco, CA, USA) and positive bands were excised from the gel using a LED-based imaging system: iBright (Thermo Fisher Scientific, A44240). The PCR products were purified from the agarose gel using the Wizard® SV Gel and PCR Clean-Up System (Promega, A9282), following the manufacturer's instructions, with the exception of using 70 °C nuclease free water for the final elution. The concentrations of purified PCR products were measured using NanoDrop.

Cloning methods have been described previously (Saghaug et al., 2020), with the only exception that *Escherichia coli* DH5α competent cells (cat nr: C2987U, NEB) were used for the transformation. Vector inserts were Sanger sequenced by Genewiz (Leipzig, Germany).

### 2.3 Data analysis of sequences and single nucleotide variation

The reference genome files of *G. lamblia* assemblage B (GS_B version 26, AHHH) were downloaded from *Giardia*db.org (Aurrecoechea et al., 2009) and imported into the genome analysis software Geneious Prime ® 2020 (Biomatter Ltd., version 2.4, Auckland, New Zealand). One FASTA-file and one general feature format (GFF) file were combined to achieve annotated reference genomes. Illumina sequenced isolates (7 previously described cultured assemblage B isolates (Saghaug et al., 2019)) were imported into Geneious and used to validate corresponding cloned sequences. Forward and reverse sequenced *gene* chromatograms from clones were aligned and compared to the reference genes of interest, *PFOR1*; GSB_114609, *PFOR2*; GSB_17063, *NR1*; GSB_22677 (*Fd-NR2*) and *NR2*; GSB_153178 (*Fd-NR1*).

For cultured, previously Illumina sequenced, isolates we validated the SNVs in the same way as described before (Saghaug et al., 2020), whereas in clinical non-cultured isolates we considered any SNV present in more than one of all clone sequences from any isolate as valid. The consensus sequences from all the clones from one isolate were aligned together and sorted into excel files with SNV positions inspired by (Lecova et al., 2019), and then sorted into haplotypes. Phylogenetic trees of the genes were made in Geneious using the Tamura-Nei with method UPGMA.

Haplotype hybrids, better known as chimeric products created during PCR amplification (Haas et al., 2011), were identified using the programs Bellerophon (Huber et al., 2004), Geneious and Excel. The consensus sequences from all the clones from one isolate were aligned and sorted into unique alleles.

### 2.4 Analysis of amino acid changes

The universal resource databank, Uniprot.org, was used to locate the ferredoxin (fd) domains. Single nucleotide variants (SNVs) were identified in Geneious. Our analysis included amino acid (aa) changes that could lead to potential rearrangements of the secondary structure, truncation of the proteins (Grantham, 1974), or aa changes located in the ferredoxin (fd) domains or in close proximity to the domain.

Open reading frames of *NR1* (GSB_22677) and *NR2* (GSB_153178) genes were modeled with simplified AlphaFold 2 (Jumper et al., 2021) using Google Colab implementation (colab.research.google.com/github/deepmind/alphafold/blob/main/notebooks/AlphaFold.ipynb), and visualized in Chimera (Pettersen et al., 2004).

## 3 Results

### 3.1 Genetic variation in clinical isolates of *Giardia*

The *PFOR1*, *PFOR2*, *NR1* and *NR2* full length genes from up to 15 recent clinical *Giardia lamblia* assemblage B isolates were successfully amplified and cloned into appropriate bacterial vector system and sequenced for characterization of single nucleotide variants (SNVs) and characterization of allelic variation. Two of the isolates failed to produce PCR products for the PFOR genes (VA and Ag10). The overall genetic variation, defined as the total number of combined SNV positions for all isolates per gene length, was similar for the NR genes: *NR1* (10.9%), and *NR2* (10.7%), while a little lower for the PFORs; 6.4% for *PFOR1* and 8.1% for *PFOR2*. The percentage of total SNV positions for individual isolates was an average of 3.5% (0.3–5.9%) for *NR1*, 2.6% (0.7–5.1%) for *NR2*, 0.2%(0–0.4%) for *PFOR1* and 0.2% (0–0.4%) for *PFOR2*. However, the higher percentage found in *NR1* is due to exceptionally high numbers of SNVs (>40) in six isolates compared to two isolates for *NR2*. The percentages of nsSNV per CDS were found to be slightly less for the *NR1* gene, 4.2% with 33 variable positions compared to 6.4% and 57 positions for the *NR2* gene (Table 2 and Supplementary Tables 3 and 4). Both PFOR genes had lower numbers of nsSNVs per gene length, where *PFOR1* was found to have 1.1% with 43 variable positions and *PFOR2* had 1.6% with 59 variable positions.

**Table 2:** Overview of *NR1*, *NR2*, *PFOR1* and *PFOR2* single nucleotide variants (SNVs) relative to the assemblage B reference genome GS_B (AHHH) and alleles.

Gene	Gene length (bp)	Number of SNV positions	SNVs per gene length (%)	Number of nsSNV positions	nsSNVs per gene length (%)	Distinct alleles found	Alleles leading to truncated proteins
*NR1*	795	87	10.9	33	4.2	69	1^a^
*NR2*	884	95	10.7	57	6.4	90	7^b^
*PFOR1*	3762	239	6.4	43	1.1	42	0
*PFOR2*	3600	293	8.1	59	1.6	70	0

a Nonsense mutation in one sample's alleles.

b Single nucleotide deletions causing frameshifts and nonsense mutations in three *Giardia* samples.

Table 3, Table 4 show that the number of SNVs per isolate in *Giardia* assemblage B NR and PFOR genes of clinical samples are variable, ranging from as low as two SNVs up to as many as 46 SNVs for the NRs and 136 for the much larger PFOR genes. SNVs and nsSNVs in the ferredoxin (fd) domains of *NR1*, *NR2*, *PFOR1* and *PFOR2* and number of identified alleles are presented in Table 3, Table 4 The *NR1* fd domains reside at positions 10–99 and 106–195, while the *NR2* fd domains are found at position 37–126 and 130–222. The fd domains of *PFOR1* are located at positions 715–749 and 778–807, while the fd domains of *PFOR2* are located at positions 697–726 and 756–790. Both cultured and non-cultured sample groups were found to contain a spectrum in the number of variants, from highly variable (reflecting potentially mixed infections), to homozygous samples. The degree of variability in *NR1* was associated with similar variability in *NR2*. For the *NR1* gene, the average number of nsSNVs per CDS length and number of nsSNVs in the fd domain were similar in the cultured and non-cultured isolates (5.3 vs 5.1 and 1.71 vs 1.63). For the *NR2* gene, a higher number of nsSNVs per CDS length and number of nsSNVs in the fd region was found in the non-cultured isolates compared to the cultured ones (14.3 vs 11.4 and 2.5 vs 1.5). The average numbers of nsSNVs for the non-cultured isolates of *PFOR1* and *PFOR2* were higher than for the cultured isolates (10.6 vs 4.1 and 6.4 vs 9.7).

**Table 3:** Number of validated cloned sequences, single nucleotide variants and alleles of *NR1* and *NR2* genes *in Giardia* assemblage B isolates.

	Nitroreductase 1					Nitroreductase 2				
	Sequenced clones^a^	SNVs	nsSNVs	nsSNVs fd	Alleles	Sequenced clones^a^	SNVs	nsSNVs	nsSNVs fd	Alleles
*Cultured samples, no clinical data available*										
P344	29	46	12	3	21	19	45	31	5	14
P387	26	42	9	4	8	14	31	16	2	5
P413	20	15	4	1	1	20	9	6	1	1
P424	19	14	3	1	1	20	9	6	1	1
P427	28	44	9	3	9	15	35	17	5	8
P433	19	2	0	0	3	16	6	2	0	4
P458	24	3	0	0	4	20	6	2	0	3
*Fecal sample, no clinical data available*										
0099	21	42	10	2	5	15	41	25	3	4
*Fecal samples, clinically susceptible*										
Ag30	16	18	1	1	1	18	6	2	0	1
VA	20	23	3	1	1	19	16	12	4^b^	1
Ag15	6	31	4	1	2	16	29	16	3^b^	3
*Fecal samples, clinically refractory*										
Ag10	16	42	8	2	4	14	28	13	2	6
Ag13	15	44	8	3	6	16	25	15	3	9
Ag20	8	25	2	1	2	6	19	10	2	3
Ag22	19	28	6	2	3	17	37	21	3	7

a The number of sequenced clones corresponds to the number of clones obtained after removal of chimeras (see Supplementary Table 2 for the total number of clones obtained).

b Single nucleotide deletions are included in the number.

**Table 4:** Number of validated cloned sequences, single nucleotide variants and alleles of *PFOR1* and *PFOR2* genes *in Giardia* assemblage B isolates.

	Sequenced clones^a^	Pyruvate ferredoxin oxidoreductase 1				Pyruvate ferredoxin oxidoreductase 2				
		SNVs	nsSNVs	nsSNVs fd	Alleles	Sequenced clones^a^	SNVs	nsSNVs	nsSNVs fd	Alleles
*Cultured samples, no clinical data available*										
P344	6	52	8		4	20	117	15	1	4
P387	13	115	16		5	19	123	10	1	14
P413	15	11	1		1	16	35	10		7
P424	19	11	1		1	14	26	1		1
P427	15	11	1		1	14	119	9		8
P433	15	12	2		2	20	0	0		1
P458	12	4	0		3	15	0	0		1
*Fecal sample, no clinical data available*										
0099	8	114	13		7	6	119	13		4
*Fecal samples, clinically susceptible*										
Ag30	7	36	5		3	7	33	4		4
VA	NA	NA	NA		NA	NA	NA	NA		NA
Ag15	11	103	10		5	11	115	8	2	6
*Fecal samples, clinically refractory*										
Ag10	NA	NA	NA		NA	NA	NA	NA		NA
Ag13	11	88	10		3	8	125	13	1	6
Ag20	4	97	14		4	16	115	8		5
Ag22	10	72	12		7	13	129	12		11

NA=Not available seuences due to not enough DNA or failure to produce PCR products from samples.

a No chimera detected for the two PFOR genes.

### 3.2 Alleles in the nitroreductase 1 and 2 genes

A total of 69 different alleles were found in the *NR1* gene, while 90 different alleles were found in the *NR2* gene in all 15 isolates of *Giardia* analyzed in the present study (Table 3). We found a total of 42 alleles for the *PFOR1* gene and 70 alleles for the *PFOR2* gene in 13 isolates. A total of three isolates (P413, P424 and P427) were found to be homozygous for PFOR1 (see Table 4). One isolate was homozygous for both PFOR genes (P424), while the two isolates P433 and P58 were homozygous for PFOR2. Four of the isolates were homozygous at both NR genes:P413, P424, VA and Ag30, Table 3. Chimeric sequences occurred for the NR genes in most of the heterozygous samples. 33 chimeric NR1 sequences were found in nine samples (range 0%–26%), and 27 NR2 chimeric sequences in ten samples (range 4%–29%) (see Supplementary Table 2). Chimeric sequences were removed from the main analysis. Even after removal of chimeras in the NR genes, the number of distinct alleles and genetic variation remained high in three cultured isolates, P344, P427 and P387, and also in the non-cultured Ag10, Ag13 and 0099 (see Table 3, Supplementary Table 2 and supplementary text 1). The number of distinct alleles was higher in the *PFOR2* gene compared to the *PFOR1* gene (see Table 4). With the number of distinct alleles exceeding four for at least one of the genes, these were considered to be possibly isolates from infections by more than one strain. For the isolates with 2–4 alleles, we found allele distributions compatible with all possible combinations (1:3, 2:2, 1:2:1, 1:1:1:1).

For the *NR1* gene, only two alleles were found to be present in more than one of the cultured isolates. The two isolates P433 and P458 had two alleles in common: one of them being identical to the reference *NR1* gene sequence, GSB_22677. No shared alleles were found in any of the non-cultured isolates.

One *NR2* allele was found to be present in three cultured isolates (P458, P344 and P433), while five other *NR2* alleles were found to be present in two of the isolates examined (see Supplementary Figs. S1 and S2 for allele distribution in phylogenetic trees). One of these alleles was found in the Ag13 isolate from a treatment refractory case and in the Ag15 isolate from a treatment-susceptible case. Another of the shared alleles was found in both a cultured isolate (P427) and a non-cultured isolate from a treatment-susceptible case (VA).

Some of the alleles from the cultured clinical isolates of *Giardia* were found in more than one isolate. For PFOR1 the two isolates P458 and P433 had the same allele as the reference sequence GSB_114609. One other allele was found to be present in the three isolates P413, P427 and P424. Two of the MTZ refractory isolates, Ag13 and Ag22 was also found to have the same *PFOR1* allele. For PFOR2 the two isolates P458 and P433 were found to have the same allele as the GSB_17063 reference. One other allele was found in the two clinical isolates P413 and P424 (see Supplementary Figs. S3 and S4).

### 3.3 *Evaluating* the putative nsSNV effects on the protein function

The number of nsSNVs found in each gene for every isolate is listed in Table 2, while the nucleotide positions and changes in the fd domain and changes causing potentially important alterations are listed in Table 5, Table 6. The rest of the SNVs, nsSNVs and deletions are presented in the Supplementary Tables 3 and 4 In NR1 a common nsSNV at codon 264 leading to a change from the basic amino acid lysine (K) to arginine (R) was found in eight of the 15 isolates (see Table 5) A nsSNV at codon 8 resulted in the large and bulky phenylalanine (F) being changed to the hydrophobic leucine (L) in the fd domain of three isolates (see Table 5). Further, a nsSNV change from the acidic aspartic acid (D) to the smallest amino acid glycine (G) at codon 101 was identified in several of the isolates from all three groups (cultured, and non-cultured isolates from MTZ susceptible and refractory cases). Isolate Ag13 was found to have one allele with a nonsense mutation at codon 195. This caused a truncated, most likely dysfunctional NR1 protein, encoded by one of the alleles of this isolate (represented by 6 out of 15 cloned sequences).

**Table 5:** Non-synonymous SNVs causing potentially important alterations in NR1, including all that were found in the ferredoxin domains.

Position Nucl. ref	10	11	17	22	40/42	59	76	88	91	145	173	302	583	786
	G	G	C	T	G-G	T	A	G	G	G	G	A	C	C
*Cultured samples, no clinical data available*														
P344			A/C				G/A				A/G	A/G		C/T
P387		C/G					G/A			A/G	A/G	A/G		C/T
P413				C										
P424				C										
P427		C/G					G/A				A/G	A/G		C/T
P433														
P458														
*Fecal sample, no clinical data available*														
0099					A-A/G-G						A/G	G		C/T
*Fecal samples, clinically susceptible*														
Ag30				C										
VA											A	G		T
Ag15											A/G	G		C/T
*Fecal samples, clinically refractory*														
Ag10	A/G										A	G		C/T
Ag13							G/A	A/G			A/G	A/G	C/T	C/T
Ag20											A			
Ag22						C/T			A/G			A/G		
Amino acid change (codon position)	G > S (4)	G > A (4)	P > Q (6)	F > L (8)	V > I (14)	V > A (20)	M > V (26)	V > M (30)	D > N (31)	G > R (49)	S > N (57)	D > G (101)	Q >* (195)	K > R(264)

**Table 6:** Non-synonymous SNVs causing potentially important alterations in NR2, including all that were found in the ferredoxin domains.

Position Nucl. ref	42	52	99	123	124	126	131	156	169	184	220	381	565	751
	T	G	C	G	A	G	G	A	T	T	A	C	A	T
*Cultured samples, no clinical data available*														
P344		A/G		T/G	G/A	G/C		C/T/A					A/G	T/C
P387					G/A			C/A					A/G	C
P413					G									
P424					G									
P427			Del		G		A/G	C		C	G/A		A/G	C
P433														T/C
P458														T/C
*Fecal sample, no clinical data available*														
0099	.	.	.	.	G/A	G/C		C/T/A						C
*Fecal samples, clinically susceptible*														
Ag30														C
VA			Del		G		A	C					G	C
Ag15	Del				G			C				Del	G	C
*Fecal samples, clinically refractory*														
Ag10					G/A			C					G	C
Ag13					G			C	C/T				A/G	C
Ag20					G			C					A/G	C
Ag22					G	G/C.		C/T					A/G	C
Amino acid change (codon position)	(14)	D > N (18)	(33)	K > N (41)	K > E (42)	K > D^a^ (42)	R > K (44)	E > D (52)	C > R^b^ (57)	S > P (62)	I > V (74)	(127)	R > G(189)	S > P(251)

a Combination of two nsSNVs with a G at position 124 and a C at position 126 changes K to D.

b Cysteine responsible for making Fe–S clusters.

For NR2, most of the samples had the nucleotide G at position 124 in the fd domain translating to glutamic acid (E) instead of the basic lysine (K) by the respective codon of the reference (see Table 6). The larger and basic arginine (R) was changed to the small glycine (G) due to an SNV in codon 189 for all three groups of samples and the aliphatic amino acid serine (S) was replaced with the ring structured proline (P) at codon position 251 in 13 out of 15 isolates. A total of seven distinct *NR2* alleles had three different single nucleotide deletions causing a frameshift mutation resulting in a nonsense mutation, hence, truncated versions of NR2. A nonsense mutation at position 33, was found in two isolates: the MTZ susceptible non-cultured isolate VA and the cultured and potentially non-clonal isolate P427 (Table 6). For the homozygous VA isolate, this means all NR2 proteins most likely would be dysfunctional. Stop codons were also found at two different positions codon 14 and 127, in two of the three alleles of the isolate Ag15, from a MTZ-susceptible case, potentially causing dysfunctionality in the majority of NR2 proteins in Ag15 (see Supplementary Table 4). In two of the alleles of *NR2* in the isolate from a MTZ-refractory case, Ag13, a nsSNV in the fd domain caused a switch from cysteine (C) to arginine (R) at codon position 57, likely disrupting this protein variant's ability to stably bind an iron-sulfur (Fe–S) cluster at this site.

For the PFOR genes, A total of four amino acid changes were found in the fd domains of the PFOR2 gene (see Table 7). The first one at codon position 241 a switch from Lysine (K) to arginine (R) where both amino acids have positively charged side chains were found in two alleles from the non-cultured isolate Ag13. In the cultured isolate P344 one allele was found to have a switch at codon 255 from the small amino acid glycine (G) to the other small amino acid cysteine (C). One allele from the MTZ-susceptible isolate Ag15 was found to have a switch from the negatively charged aspartic acid (D) to the uncharged asparagine at codon 262. Finally, a switch from the hydrophobic alanine (A) to the polar and uncharged threonine at codon 264 was found in seven alleles of the cultured isolate P387. The nsSNVs outside of the fd domains were mostly conservative, meaning that important properties of the substitute amino acids were not changed (see Supplementary Tables 5 and 6 for overview).

**Table 7:** Non-synonymous SNVs causing potentially important alterations in PFOR2 in the ferredoxin domains.

Position Nucl. ref	722	763	784	790
	A	G	G	G
*Cultured samples, no clinical data available*				
P344		G/T		
P387				G/A
P413				
P424				
P427				
P433				
P458				
*Fecal sample, no clinical data available*				
0099	.	.	.	.
*Fecal samples, clinically susceptible*				
Ag30				
VA				
Ag15			G/A	
*Fecal samples, clinically refractory*				
Ag10				
Ag13	G/A			
Ag20				
Ag22				
Amino acid change (codon position)	K > R(241)	G > C (255)	D > N (262)	A > T (264)

To gain further insight into the potential importance of different mutations and their locations in the NR1 and NR2 subdomains, we constructed models using AlphaFold 2 and mapped the identified nsSNV in these (Fig. 1). *Giardia* NR1 and 2 have a unique domain organization with an fd domain and NR domain together. No structural homologs exist with the same domain organization, based on a sequence search in Protein Data Bank. Some segments within the models score high, and these are interspersed with low scoring segments.

**Fig. 1:**
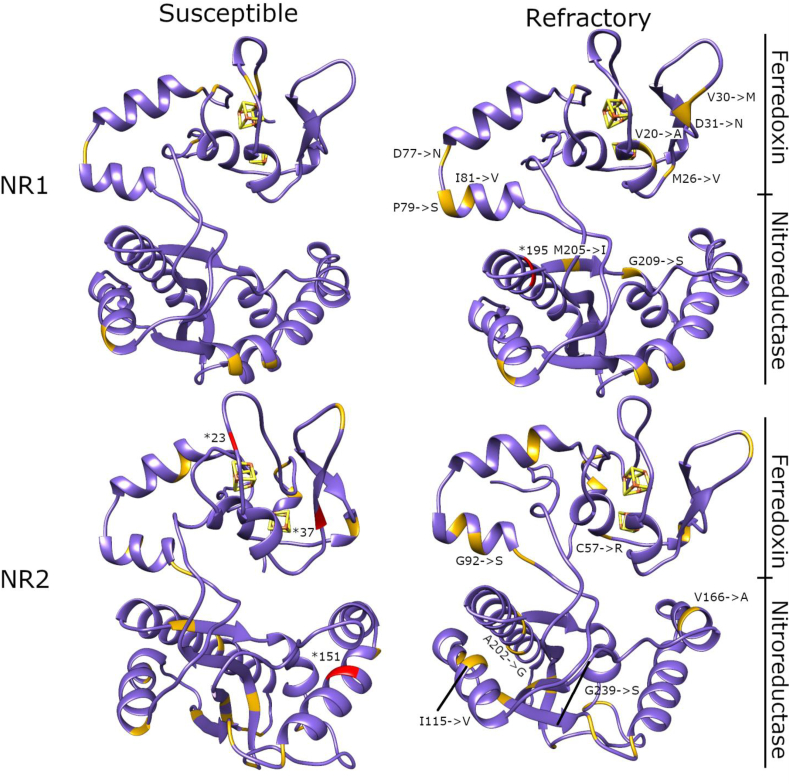
Cartoon presentations of NR1 and NR2 homology models. The ferredoxin (fd) domain is above, and iron-sulfur cluster is shown as a red-yellow cage within the fd domain. The larger NR domain is below. nsSNVs identified in the present study are depicted with orange color. Red color indicates nonsense mutations at positions 23, 37 and 151 from susceptible NR2 isolates, and a nonsense mutation leading to a premature stop codon at position 195 from a refractory NR1 isolate (positions highlighted by *). (For interpretation of the references to color in this figure legend, the reader is referred to the Web version of this article.)

First, we compared our homology model to other structurally related NRs and fd containing proteins using the DALI server (Holm, 2020). Structural sequence alignments showed that both subdomains had conserved residues, and these were also retained in our homology models. This suggests that nsSNVs do not obstruct activity of NR1 and NR2. nsSNVs unique to clinical isolates from treatment refractory cases in NR1 were mostly found in the fd domain (Fig. 1). Mutations in NR2 refractory and susceptible samples are mostly in overlapping positions. Unlike in NR1, mutations in NR2 are spatially more distributed along the protein, and we found only one mutation in the fd domain.

## 4 Discussion

In the present study, we were able to investigate in a select collection of non-cultured clinical samples of both, MTZ susceptible and refractory *Giardia* assemblage B isolates, and axenized isolates derived by limiting dilution, the genetic variation and allelic diversity of the four full length MTZ-metabolism genes, *NR1*,*NR2, PFOR1* and *PFOR2*.

### 4.1 Genetic variation and alleles of the genes

For some of the non-cultured isolates, such as Ag20 and Ag15, we obtained a limited number of sequences for both of the NR genes. The number of alleles found in these two isolates must be interpreted with caution.

The number of distinct alleles and genetic variation was high in the NR genes for three of the cultured isolates, P344, P427 and P387 and also in the non-cultured samples Ag10 and Ag13 (Table 3). The number of distinct alleles was also high for PFOR genes where P387, Ag15, Ag22 had more than four alleles. The presence a high number of distinct alleles could indicate that these isolates potentially could represent non-clonal isolates. This is puzzling since the cultured isolates were originally derived by limiting dilution. There is a possibility that they are co-existing lineages with some interdependency, or some new mutations could have occurred to adapt to in vitro culture, or a combination of both. For the isolate with the highest number of distinct alleles for *NR1*, P344, a total of 36 clones were sequenced and 21 alleles were found, while 20 clones were obtained for *NR2* resulting in 14 alleles (see Supplementary Table 2). For the *PFOR2* gene from the isolate P387 19 clones were sequenced and a total of 14 distinct alleles were identified. Several studies have concluded that the presence of more than four alleles in single isolates most likely are due to mixed infections or perhaps intragenic recombination (Lalle et al., 2005; Kosuwin et al., 2010; Siripattanapipong et al., 2011; Waldram et al., 2017; Lecova et al., 2019). This may happen if an infected host is subsequently reinfected, with a different *Giardia* strain (Sprong et al., 2009). An isolate may still originate from one clone but be unstable and generate many variants. While the number of isolates examined in this study is small, especially for the PFOR genes of non-cultured isolates, we nevertheless noted that only one of the four isolates, Ag22 for the *PFOR1* gene, from treatment-refractory cases was homozygous, and two of the non-cultured MTZ susceptible samples were homozygous for the NR1 and NR2 loci. One of the samples that were homogenous for both of the NR genes, VA, is a sample obtained from the Bergen outbreak in 2004. A previous study investigated the sequences of glutamate dehydrogenase (gdh) and beta-giardin (Robertson et al., 2007). It was observed that some of the gene sequences found to be non-dominant in the beginning of the study were predominating at a later time point. The authors suggest that these sequences may be associated with a higher potential of transmission including virulence, environmental resistance, increased proliferation, or even a combination of factors.

Whether homozygous or heterozygous NR and PFOR alleles may decrease or increase resilience against MTZ, should be assessed in further studies.

### 4.2 Putative effect of SNV induced mutations

Several enzymes are involved in the activation of MTZ, and several metabolic systems may be involved in protection against the reactive toxic metabolites and contribute to resistance (Leitsch, 2019). The MTZ resistance mechanisms in *Giardia* is likely pleiotropic and may be a combination of several aspects such as a resistance mechanism where MTZ-activating enzymes become downregulated, or MTZ inactivating enzymes become up-regulated, and/or genetic variation and post-transcriptional changes (Ansell et al., 2015, 2017; Leitsch, 2017; Müller et al., 2018). Possibly, different *Giardia* strains may evolve distinct resistance strategies that result from combinations of these mechanisms.

There are at least three different enzymes, NR1, PFOR1 (possibly PFOR2) and thioredoxin reductase that contribute to the activation of MTZ in *Giardia*, whereas only one enzyme, NR2, has been associated with inactivation. If the parasite is either producing less NR2 (downregulation of the gene itself) or the NR2 is dysfunctional, the parasite would most likely be more susceptible to MTZ. In the non-cultured samples for which clinical information was available for all patients, we found two susceptible samples with frameshift mutations, leading to potentially truncated and dysfunctional NR2 proteins. Interestingly, one of the susceptible isolates (VA) was homozygous, indicating viability without NR2 function. There may be redundancy in NR2 function, allowing higher tolerance for genetic variability.

Mutations that would make the NR1 enzyme less efficient at activating MTZ or dysfunctional, could lead to reduced levels of toxic metabolites of the drug, and make the parasite more tolerant to MTZ. A study by Ansell et al., (2017) described the same nonsense mutation at nucleotide position 583 (codon position 195) as identified in our study. This mutation was found in some transcripts of NR1 in the laboratory induced MTZ-resistant isolate, 106-MtzR (Ansell et al., 2017). Further, two studies have reported that NR1 was downregulated in three resistant *Giardia* strains (Ansell et al., 2017; Emery et al., 2018), supporting the notion that a dysfunctional NR1 could be protective against MTZ, as seen in the refractory Ag13 sample in the present study. Indeed, mutational inactivation of a gene coding for a NR, rdxA, in *Helicobacter pylori* has previously been associated with resistance towards MTZ (Goodwin et al., 1998; Jenks and Edwards, 2002). Additionally, mutations and a nonsense mutation have been found in the two NR genes *ntr4*_*TV*_ and *ntr6*_*TV*_ of MTZ resistant *T. vaginalis* (Paulish-Miller et al., 2014). Nonsense mutations should therefore be further explored in samples from clinically MTZ-refractory *Giardia* infections to assess whether these mutations could be potential resistance markers.

It has been observed that laboratory strains of WB *Giardia* may lose its resistance during one en-/excystation cycle (Muller et al., 2008). This loss of resistance was found be relevant for the two drugs MTZ and nitazoxanide, and could potentially mean that resistance may not be carried through generations. Still, the authors debate whether this could be due to factors such as acquired mutations rendering the cells unable to encyst or that encystation may have eliminated the resistant population or selective pressure may have affected the resistant cells in a negative manner. This may as well mean that the drug resistant cells were not able to grow properly in the new environment after encystation. Loss of resistance during cell cycles may be linked to epigenetic and post-transcriptional changes than specific mutations being responsible for this temporary resistance pattern (Loderstädt and Frickmann, 2021). MTZ is most likely activated through several different pathways and will therefore exhibit a pleiotropic mode of action (Holmes et al., 2016). The resistance could also manifest differently in laboratory strains than in clinical isolates of *Giardia.* The mutations and deletions found in our study should therefore be tested *in vivo* to properly understand whether potential markers of resistance may be hereditary. It is clear that further research is needed for understanding how *Giardia* may pass on resistance.

Both NR and PFOR proteins contain a fd domain. The fd domain contains cysteine residues with iron-sulfur (Fe–S) cluster forming abilities which are part of redox-active centers responsible for biological electron transport (Johnson et al., 2005; Ansell et al., 2017). One of the cysteines in NR2 was changed to an arginine in the fd domain in the refractory isolate Ag13. However, this refractory isolate had a total of nine distinct alleles, probably due to a mixed infection, making the effect of this mutation difficult to interpret. In the PFOR2 an arginine was found to be switched to a cysteine at codon 255 in the cultured clinical isolate P344. This extra cysteine could potentially affect the electron transfer abilities of the domain. We also observed a switch from the negatively charged aspartic acid (D) to the uncharged asparagine at codon 262 for the MTZ-susceptible isolate Ag15, which potentially could influence the domain and even so the MTZ metabolizing capacity of the PFOR2. One other switch that could have impact on the fd domain of PFOR2 is the change of the hydrophobic alanine (A) to the polar and uncharged threonine at codon 264 found in alleles of the cultured isolate P387. Further research is needed to interpret how amino acid changes within the fd domains potentially can alter the function and MTZ metabolizing capacities of the proteins.

### 4.3 Strengths and limitations

A strength of the current study is the inclusion of non-cultured *Giardia* samples from relatively recent clinical patient samples. The reference strains used in most other laboratory MTZ resistance studies were isolated decades ago. The MTZ tolerance slowly induced in these isolates may not reflect the mechanisms behind the increase in MTZ treatment refractory infections observed in recent years (Munoz Gutierrez et al., 2013; Nabarro et al., 2015; Cañete et al., 2020).

For the three genes, *NR2*, *PFOR1* ane *PFOR2*, we found presence of more nsSNVs in the non-cultured isolates compared to the cultured ones (see Table 3, Table 4). We cannot exclude that this could be due to mixed infections as they were not subject to the same limiting dilution procedures done for the cultured isolates.

Since no specific resistance mechanism has yet been discovered in *Giardia*, and treatment failure can result not only from resistant isolates, but also immune deficiency, poor treatment compliance and reinfection (Nash et al., 2001), the term refractory is used here to refer to treatment failure. A further strength of this study is that no signs of immunodeficiency or poor drug compliance were present in the cases with MTZ treatment refractory infections, and, because the risk of reinfection is small in Norway, these factors can be discounted as confounders and, as a consequence, the clinically MTZ refractory presentation is likely linked to the *Giardia* genotype. These refractory cases were later cured using secondary or tertiary drug regimens according to Morch et al., 2008) (Morch et al., 2008), with a lab confirmed *Giardia* negative stool sample after successful treatment.

A limitation was that some of the collected clinical samples could not be included or obtained in the study, especially for the two PFOR genes where only 4–6 clones were obtained from some of the isolates, due to low *Giardia* DNA content, making the total number of susceptible and refractory samples low, and the results need to be interpreted with caution. We also noted that the quality sequenced PFOR clones varied, even if a high number of clones was selected for sequencing, As both of the PFOR genes are 3–4 times bigger than the NR genes, several sequencing primers are needed (5–6). If one or more of these primers fail to produce a sequence, the clone cannot be analyzed. Samples with low concentrations of DNA may be analyzed in future studies by doing multiple IMS purifications and combining them before DNA extraction is carried out.

Errors induced by many amplification cycles should also be noted as a potential limitation, even if several measures were taken to avoid them (i.e., using high fidelity polymerase). It is our experience that designing and optimizing specific primers help lower the number of amplification cycles.

The eventual importance of the nsSNVs identified in this study needs to be explored in functional studies. Expression of the identified alleles could be tested *in vitro* (Müller et al., 2021) or *in vivo* in transformed *E. coli* susceptibility assays such as agar disk diffusion assays, as previously demonstrated by Müller et al. (Muller et al., 2007, 2013, 2015; Muller and Muller, 2019), or in a model system of genetically modified *Giardia* trophozoites (Jex et al., 2020). Although SNVs in full-length genes were analyzed in the present study, SNVs in potential promoter regions or before/after the CDS could potentially affect transcription of NR1 and NR2 and could be analyzed in future studies.

### 4.4 Conclusion

The considerable genetic variation in the two MTZ-metabolizing genes *NR1* and *NR2* described in this collection of *Giardia* assemblage B samples show the potential for genetic alterations affecting MTZ susceptibility and resistance. Frameshift mutations potentially leading to truncated NR2 proteins in susceptible *Giardia* isolates may affect MTZ tolerance in the parasite, as NR2 is important for detoxification of MTZ. On the other hand, a nonsense mutation in NR1, possibly leading to a truncated enzyme in a refractory *Giardia* isolate may protect the parasite due to NR1 MTZ-activating capabilities. Further, amino acid changes found in the ferredoxin domains may alter protein function and possibly help mediate increased MTZ tolerance.

We identifed a total of three nsSNVs in the ferredoxin domain of PFOR2 that potentially could affect the electron transfer capacity of the protein. The PFOR genes were found to be more conserved with fewer nsSNVs per gene length and generally fewer distinct alleles than the NR genes.

## Ethical aspects

No patient data was collected and used from the RKI laboratory cultured isolates presented in the current study; any link between individual parasite data and patient information was removed before WGS of trophozoites and cloning experiments were carried out. For the eight non-cultured clinical isolates obtained from Haukeland University Hospital, the study was approved by the Regional Committee for Medical Research Ethics (REC) of Western Norway (2013/1285/REK vest). The VA sample was obtained from the combined research and clinical biobank of the infectious diseases department (REK vest 165.04) (Infeksjonsseksjonenes kombinerte forskning-og kliniske biobank).

## Data availability

The sequences for the identified *NR1*, *NR2*, *PFOR1* and *PFOR2* alleles have been submitted to Genbank and have the following accession numbers: BankIt2664166: OQ267781-OQ268066.

## Funding

This study has been funded by a grant from the Norwegian Surveillance System for Antimicrobial Drug Resistance (NORM), a grant from the 10.13039/501100005036Centre for Pharmacy, University of Bergen, a grant from Helse-Vest (grant number 912245) and The 10.13039/100014252National Graduate School in Infection Biology and Antimicrobials (IBA). All of the experiments, data analysis and evaluation of the results in the present study were performed independently by the authors, without any interference from any of the funding institutions.

## Declaration of competing interest

No conflict of interest has been reported by any of the authors or funding institutions.

## Figures and Tables

**figs1:**
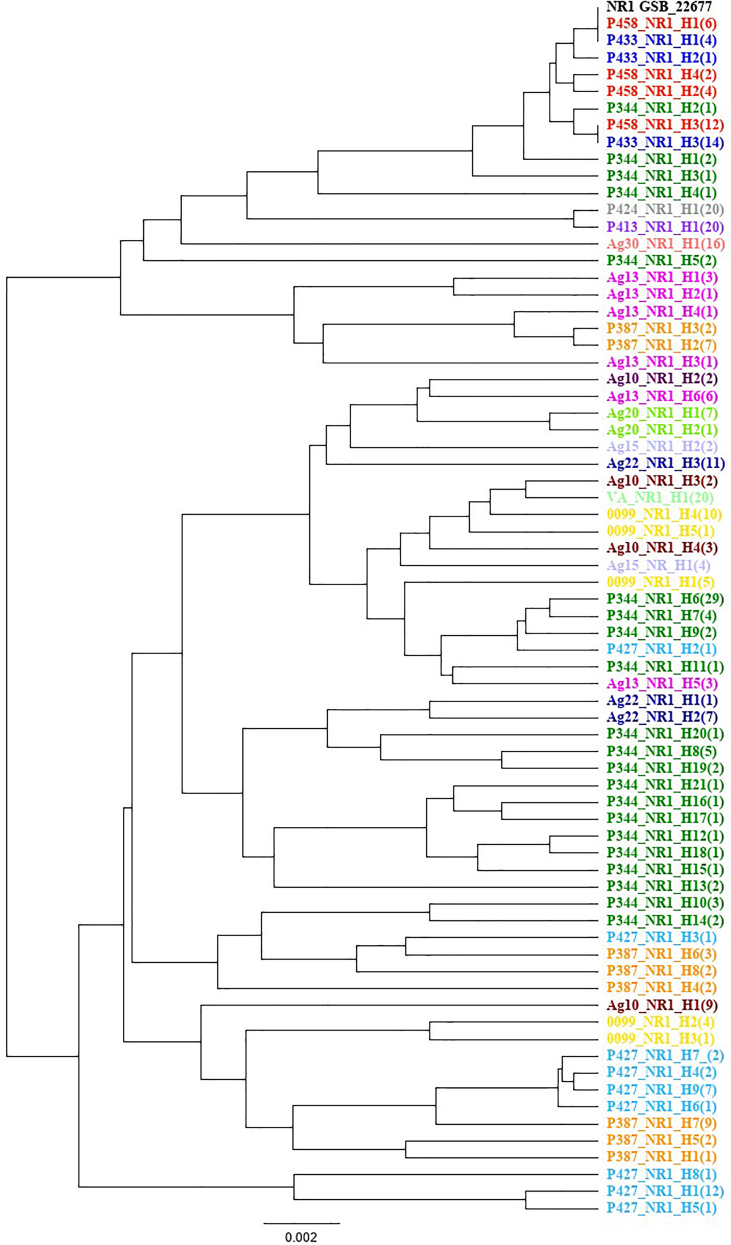


**figs2:**
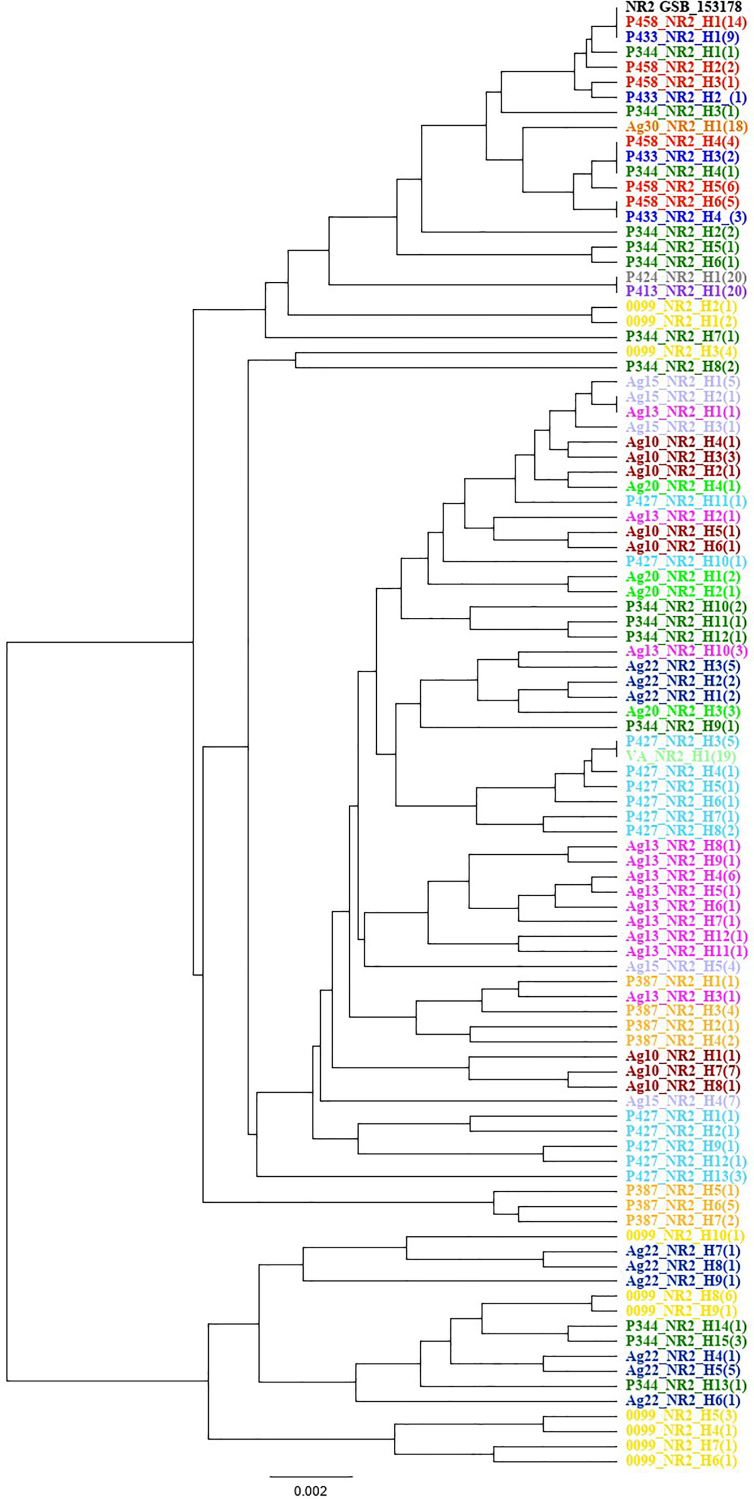


**figs3:**
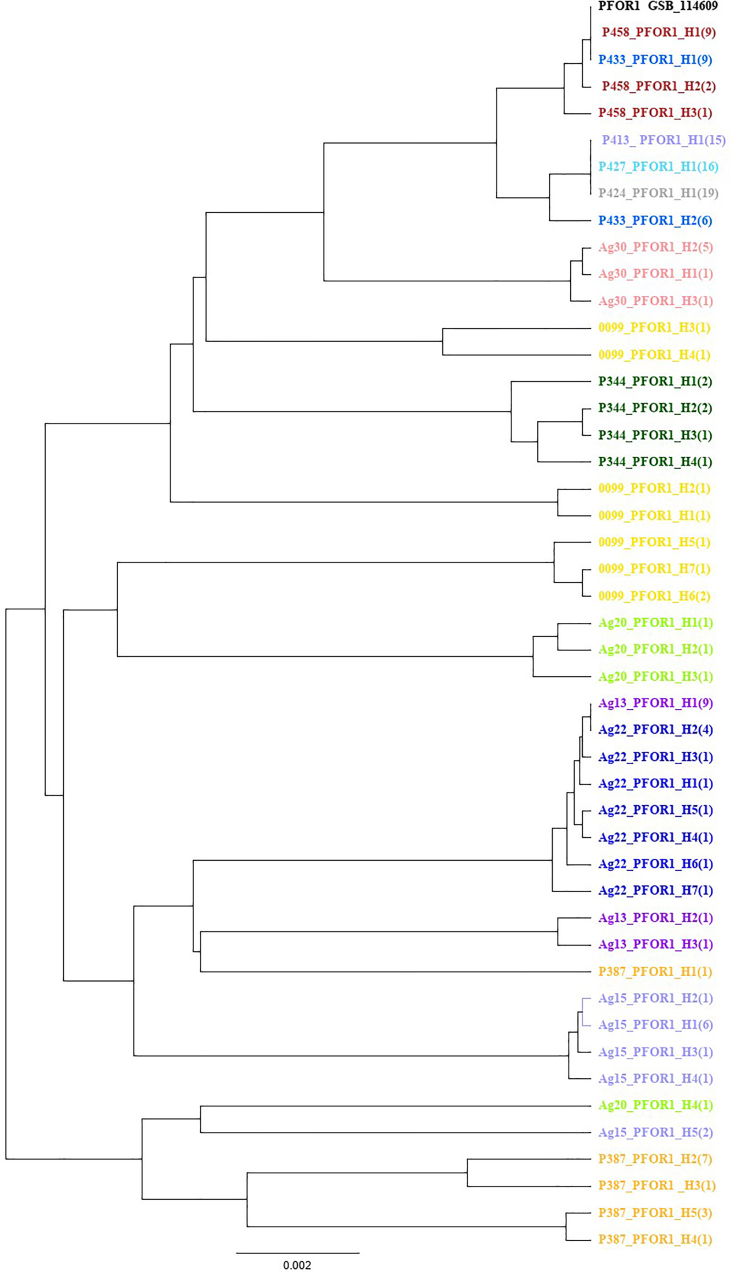


**figs4:**