# Supplementary material for: Genetic diversity in the metronidazole metabolism genes nitroreductases and pyruvate ferredoxin oxidoreductases in susceptible and refractory clinical samples of Giardia lamblia
Source: Int J Parasitol Drugs Drug Resist. 2022 Dec 21;21:51–60. doi: 10.1016/j.ijpddr.2022.12.003 (PMC9871439; doi:10.1016/j.ijpddr.2022.12.003)
Supplement: Multimedia component 1 [file mmc1.docx]

**Supplementary files**

**Genetic diversity in the metronidazole metabolism genes nitroreductase s and pyruvate ferredoxin oxidoreductases in susceptible and refractory clinical samples of *Giardia lamblia***

Christina Saghaug^1,2^, Astrid L. Gamlem^1^, Juha Vahokoski^1,3^, Christian Klotz^4^, Toni Aebischer^4^, Nina Langeland^1,2^ and Kurt Hanevik^1,2^

^1^Department of Clinical Science, University of Bergen, Bergen, Norway.
^2^Norwegian National Advisory Unit on Tropical Infectious Diseases, Department of Medicine, Haukeland University Hospital, Bergen, Norway.
^3^Department of Medicine, Haukeland University Hospital, Bergen, Norway. ^4^Department of Infectious Diseases, Unit 16 Mycotic and Parasitic Agents and Mycobacteria, Robert Koch-Institute, Berlin, Germany. **Supplementary** **Table 1: Available clinical isolates of *Giardia* assemblage B, year of collection and type of sample**

| Isolate | Year of collection | Type of sample | Treatment status |
| --- | --- | --- | --- |
| P344 | 2013 | Clinical cultured sample | NA |
| P387 | 2013 | Clinical cultured sample | NA |
| P413 | 2014 | Clinical cultured sample | NA |
| P424 | 2014 | Clinical cultured sample | NA |
| P427 | 2014 | Clinical cultured sample | NA |
| P433 | 2014 | Clinical cultured sample | NA |
| P458 | 2014 | Clinical cultured sample | NA |
| 0099 | 2014 | Clinical non-cultured sample | NA |
| Ag15 | 2013 | Clinical non-cultured sample | Susceptible |
| Ag29 | 2014 | Clinical non-cultured sample | Susceptible |
| Ag30 | 2017 | Clinical non-cultured sample | Susceptible |
| Hus25 | 2017 | Clinical non-cultured sample | Susceptible |
| Hus27 | 2017 | Clinical non-cultured sample | Susceptible |
| VA | 2004 | Clinical non-cultured sample | Susceptible |
| GB | 2004 | Clinical non-cultured sample | Susceptible |
| Ag10 | 2013 | Clinical non-cultured sample | Refractory |
| Ag13 | 2013 | Clinical non-cultured sample | Refractory |
| Ag20 | 2014 | Clinical non-cultured sample | Refractory |
| Ag22 | 2014 | Clinical non-cultured sample | Refractory |

^1^Samples taken before MTZ treatment
NA= Not applicable

**Supplementary text 1: Chimeric sequences**
Chimeric sequences were in particularly a challenge in heterozygous and mixed samples. In a recent study where the sequencing methods Illumina and Sanger were both used to explore mixed *Giardia* infections, up to 20 % of the Illumina sequences were filtered out of the analysis due to chimeric features.(Maloney, Molokin et al. 2020) A high number of cycles in the gene amplification PCR results in more chimeric sequences, and Wang and Wang noticed already in 1996 a 30% increase in chimeras when the cycles of the PCR was above 30 (Wang and Wang 1996). In order to achieve high enough concentrations of PCR products, we and others have often used more than 30 amplification cycles(Lasek-Nesselquist, Welch et al. 2009, Kosuwin, Putaporntip et al. 2010, Siripattanapipong, Leelayoova et al. 2011, Mizuno, Matey et al. 2020).
 Nevertheless, we did identify homozygous alleles of the two *NR* genes in some of the isolates, such as P424, with identical sequences in 19 clones of *NR1* and only one low confidence SNV in one of 20 *NR2* clones. This provides evidence that the method is suitable for obtaining specific and correct sequences. No chimeric sequences were found for the two PFOR genes.

**Supplementary Table 2: Total numbers of sequenced clones, percentage of chimeras and number of identified chimeras in the NR genes**

|  |  |  |  |  |  |  |  |
| --- | --- | --- | --- | --- | --- | --- | --- |
|  | **Nitroreductase 1** | | |  | **Nitroreductase 2** | | |
|  | Sequenced clones | Percentage chimera | Number of chimeric sequences |  | Sequenced clones | Percentage chimera | Number of chimeric sequences |
| *Cultured samples, no clinical data available* | | | | | | | |
| P344 | 36 | 19 | 7 |  | 20 | 5 | 1 |
| P387 | 39 | 26 | 11 |  | 16 | 12 | 2 |
| P413 | 20 | 0 | 0 |  | 20 | 0 | 0 |
| P424 | 19 | 0 | 0 |  | 20 | 0 | 0 |
| P427 | 33 | 15 | 5 |  | 20 | 25 | 5 |
| P433 | 21 | 10 | 1 |  | 16 | 0 | 0 |
| P458 | 25 | 4 | 1 |  | 23 | 14 | 3 |
| *Fecal sample, no clinical data available* | | | | | | | |
| 0099 | 24 | 12.5 | 3 |  | 21 | 29 | 6 |
| *Fecal samples, clinically susceptible* | | | | | | | |
| Ag30 | 16 | 0 | 0 |  | 18 | 0 | 0 |
| VA | 20 | 0 | 0 |  | 19 | 0 | 0 |
| Ag15 | 6 | 0 | 0 |  | 18 | 11 | 2 |
| *Fecal samples, clinically refractory* | | | | | | | |
| Ag10 | 17 | 6 | 1 |  | 16 | 13 | 2 |
| Ag13 | 19 | 21 | 4 |  | 19 | 16 | 3 |
| Ag20 | 8 | 0 | 0 |  | 7 | 14 | 1 |
| Ag22 | 20 | 5 | 1 |  | 19 | 11 | 2 |

**Figure S1: Phylogenetic tree of NR1 alleles**.


**Figure S2:** **Phylogenetic tree of NR2 alleles**.

 **Figure S3: Phylogenetic tree of PFOR1 alleles**.


**Figure S4:** **Phylogenetic tree of PFOR alleles**.

**References**

Kosuwin, R., C. Putaporntip, U. Pattanawong and S. Jongwutiwes (2010). "Clonal diversity in Giardia duodenalis isolates from Thailand: evidences for intragenic recombination and purifying selection at the beta giardin locus." Gene **449**(1-2): 1-8.

Lasek-Nesselquist, E., D. M. Welch, R. C. Thompson, R. F. Steuart and M. L. Sogin (2009). "Genetic exchange within and between assemblages of Giardia duodenalis." J Eukaryot Microbiol **56**(6): 504-518.

Maloney, J. G., A. Molokin and M. Santin (2020). "Assessment of next generation amplicon sequencing of the beta-giardin gene for the detection of Giardia duodenalis assemblages and mixed infections." Food Waterborne Parasitol **21**: e00098.

Mizuno, T., E. J. Matey, X. Bi, E. M. Songok, H. Ichimura and M. Tokoro (2020). "Extremely diversified haplotypes observed among assemblage B population of Giardia intestinalis in Kenya." Parasitol Int **75**: 102038.

Siripattanapipong, S., S. Leelayoova, M. Mungthin, R. C. Thompson, P. Boontanom, W. Saksirisampant and P. Tan-Ariya (2011). "Clonal diversity of the glutamate dehydrogenase gene in Giardia duodenalis from Thai isolates: evidence of genetic exchange or mixed infections?" BMC Microbiol **11**: 206.

Wang, G. C. Y. and Y. Wang (1996). "The frequency of chimeric molecules as a consequence of PCR co-amplification of 16S rRNA genes from different bacterial species." Microbiology (Reading) **142 ( Pt 5)**: 1107-1114.
